# IgG4 Autoimmune Disease Masquerading As Oroantral Fistula

**DOI:** 10.7759/cureus.42475

**Published:** 2023-07-26

**Authors:** Patrik Schmidt, Abeer Qasim, Husnain R Ali, Vedangkumar Bhatt, Muhammad Sulh, Misbahuddin Khaja, Kalpana A Uday

**Affiliations:** 1 Internal Medicine, BronxCare Health System, Bronx, USA; 2 Medicine, American University of the Caribbean, New York City, USA; 3 Pathology and Laboratory Medicine, BronxCare Health System, Bronx, USA; 4 Internal Medicine/Pulmonary Critical Care, Icahn School of Medicine at Mount Sinai/Bronx Care Health System, Bronx, USA; 5 Internal Medicine, Icahn School of Medicine at Mount Sinai, New York City, USA; 6 Nephrology, BronxCare Health System, Bronx, USA

**Keywords:** unusal presentaion of igg-4 autoimmune disease, oroantral fistula revealing igg4 autoimmune disease, rare manefestations of igg-4 related disease, igg4-related disease, igg4 autoimmune disease

## Abstract

IgG4-related disease (IgG4-RD) is an immune-mediated disorder that involves multiple organs and is characterized by the infiltration of lymphoplasmacytic cells, including IgG4-positive plasma cells, along with storiform fibrosis and obliterative phlebitis in the inflamed organs. The primary sites affected by this condition include the pancreas, bile ducts, salivary glands, aorta, lungs, kidneys, meninges, lacrimal glands, mediastinal lymph nodes, and retroperitoneum. The pathogenesis is linked to a type 2 T-helper-cell cytokine profile and the involvement of regulatory T cells. However, the exact mechanism is still unknown. Patients with IgG4-related disease are frequently misdiagnosed as having malignancies due to the resemblance of the lesions to infections or other immune-mediated diseases and certain tumors, such as pancreatic cancer and pseudo-renal pelvis tumor. Prompt identification of IgG4-related disease is essential as a delayed diagnosis until advanced stages can result in severe organ damage and potentially fatal outcomes, despite the disease being highly responsive to treatment. This report presents a highly unusual case of IgG4-related disease (IgG4-RD) with an atypical presentation in a 38-year-old female patient. The patient sought medical attention in the emergency department due to nasal septal erosions and an oral-antral fistula. Nasal cultures were conducted and indicated the presence of Klebsiella ozaena. Subsequent investigations, including a nasal biopsy, confirmed the diagnosis of IgG4-related autoimmune disease.

## Introduction

IgG4-related disease, also known as IgG4-related systemic disease or IgG4-RD, is a chronic autoimmune disorder that can affect various organs and tissues in the body. It is characterized by the infiltration of immune cells, primarily plasma cells, and the accumulation of IgG4 antibodies in the affected tissues. Elevated levels of IgG4 in the bloodstream distinguish IgG4-related diseases, and the presence of sclerosing inflammation is accompanied by a significant number of positive plasma cells for IgG4 [1]. The prevalence rate of IgG4-related disease is approximately 2.2 cases per 100,000 individuals [2]. In terms of epidemiology, there seems to be a higher susceptibility to IgG4-related disease among Asian populations compared to other ethnic groups [3]. IgG4 antibodies can be detected in various medical conditions, including helminth infections, allergies, cancer, and rheumatoid arthritis. They now constitute a newly recognized category of illness characterized by the presence of pathogenic, antigen-specific autoantibodies belonging to the IgG4 subclass [4]. Diagnosing IgG4-related disease (IgG4-RD) is challenging and involves conducting a clinical assessment, radiology, analyzing tissue samples under a microscope, utilizing imaging techniques, and performing blood tests to assess specific antibodies [5]. However, the gold standard is tissue biopsy. Here, we present a case of a 38-year-old female who initially presented with nasal septal perforation and an oral-antral fistula and underwent a nasal cavity biopsy that confirmed the diagnosis of IgG4-related disease. 

## Case presentation

A 38-year-old woman arrived at the emergency department with complaints of progressive headaches, an observed cavity in her hard palate, and clear nasal discharge for five days. Her headaches were constant, affecting both sides of the head and resembling migraines, but without accompanying nausea and vomiting or focal neurological deficits. The opening in the roof of her mouth caused her discomfort when drinking liquids, leading to regurgitation through the nostrils. She also reported an altered ability to smell. She denies experiencing any pain in her nose or facial/maxillary region. There is no history of trauma, except for a full dental extraction she underwent two to three years ago. She mentions having a chronic scab over her upper right molars that has remained unchanged and painless. She has a past medical history of hyperthyroidism, hypocalcemia, chronic kidney disease, migraine, depression, and rheumatic fever. She also had a full dental extraction two to three years ago. Her home medications include benazepril, bupropion, zolpidem, paroxetine, buspirone, propranolol, and methimazole. She has a smoking history of half a pack of cigarettes per day, but denies alcohol use, and has a family history of diabetes on both her paternal and maternal sides. She also denied any drug use or any significant family history. 

Upon arrival at the emergency department, her vital signs were stable with a blood pressure of 133/57 mmHg, a temperature of 98.6 degrees F, a pulse of 94 BPM, and a SpO2 of 100% on room air. On physical examination, a minor hole measuring less than 1 centimeter was observed in the hard palate, located at the center of the roof of the mouth. The remaining aspects of the examination were unremarkable. 

Her initial laboratory results indicated the presence of microcytic anemia, with a hemoglobin/hematocrit (Hb/Hct) ratio of 9.4/30% and a mean corpuscular volume (MCV) of 69.6. Additionally, the CRP level was elevated at 23.52 mg/dL, while the remaining laboratory values fell within normal ranges. Please refer to Table 1. 

**Table 1:** Presenting Lab Values. WBC: white blood cells, RBC: red blood cells, MCV: mean corpuscular volume, HGB: hemoglobin, CRP: C-reactive protein

Labs	Patient's lab	Normal ranges
WBC Count	4.2(L)	4.8-10.8 k/ul
RBC Count	4.31	4.00-5.20 MIL/ul
MCV	69.6(L)	80.0-96.0 fL
HGB	9.4(L)	12.0-16.0 g/dl
Hematocrit, Whole Blood	30.0(L)	42.0-51.0 %
Eosinophil %	0.0	<=5.0- %
Blood Urea Nitrogen, Serum	11.0	6.0-20.0 mg/dL
Creatinine, Serum	0.5	0.5-1.5 mg/dL
Calcium, Total Serum	9.1	8.5-10.5 mg/dL
Sodium, Serum	142	135-145 mEq/L
Potassium, Serum	3.4(L)	3.5-5.0 mEq/L
CRP	23.52 mg/dL	<5 mg/dl
IgG4 levels	150.8mg/dl	4-86mg/dl

**Figure 1:**
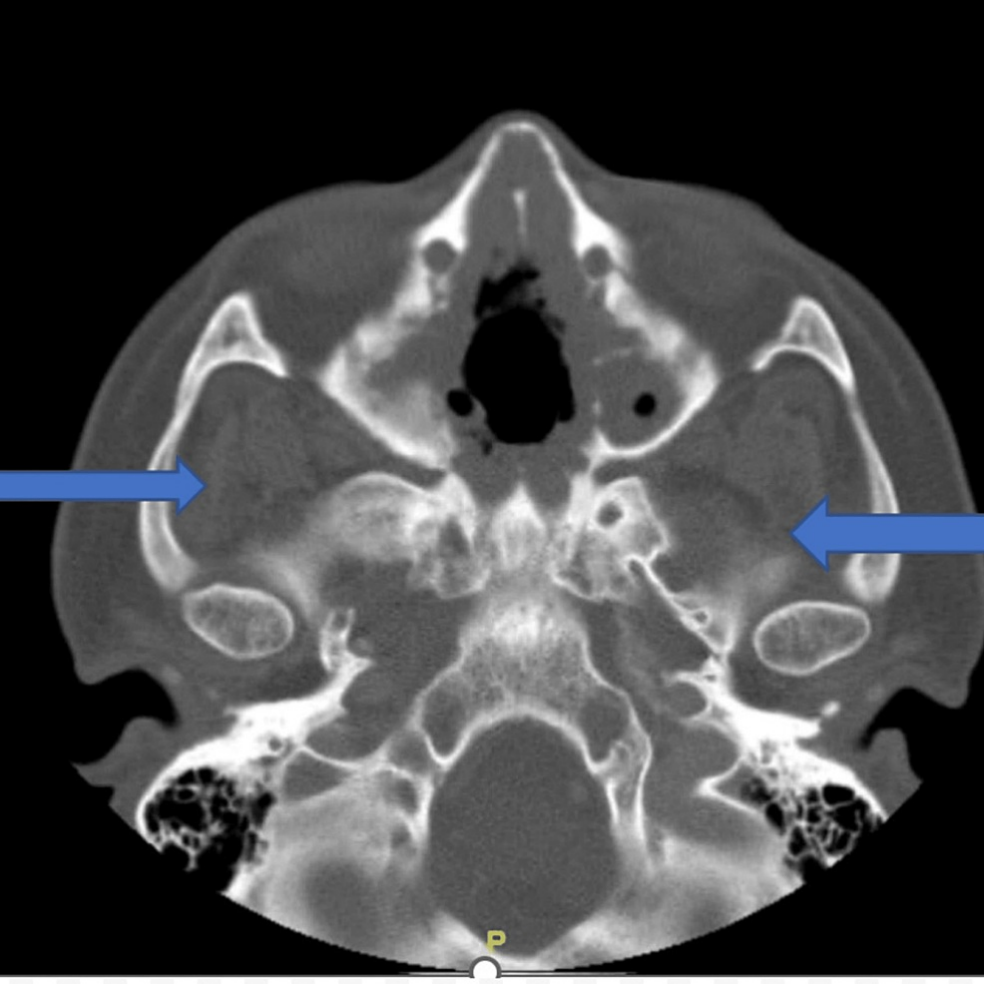
There is an irregular appearance of the medial bilateral maxillary sinus walls as well as the nasal septum suggesting resorptive process (see arrows). Diffuse thickening of the maxilla. No rim-enhancing fluid collection is identified. No focal enhancing soft tissue lesion is identified.

As a result of the signs and symptoms, a CT scan of sinuses with contrast was ordered, which revealed the complete opacification of the maxillary, sphenoid sinuses, and anterior ethmoid cells and partial opacification of the bilateral frontal sinuses. The rest of the findings are described in Figure 1.

CT Scan of the head was negative for any acute findings

**Figure 2:**
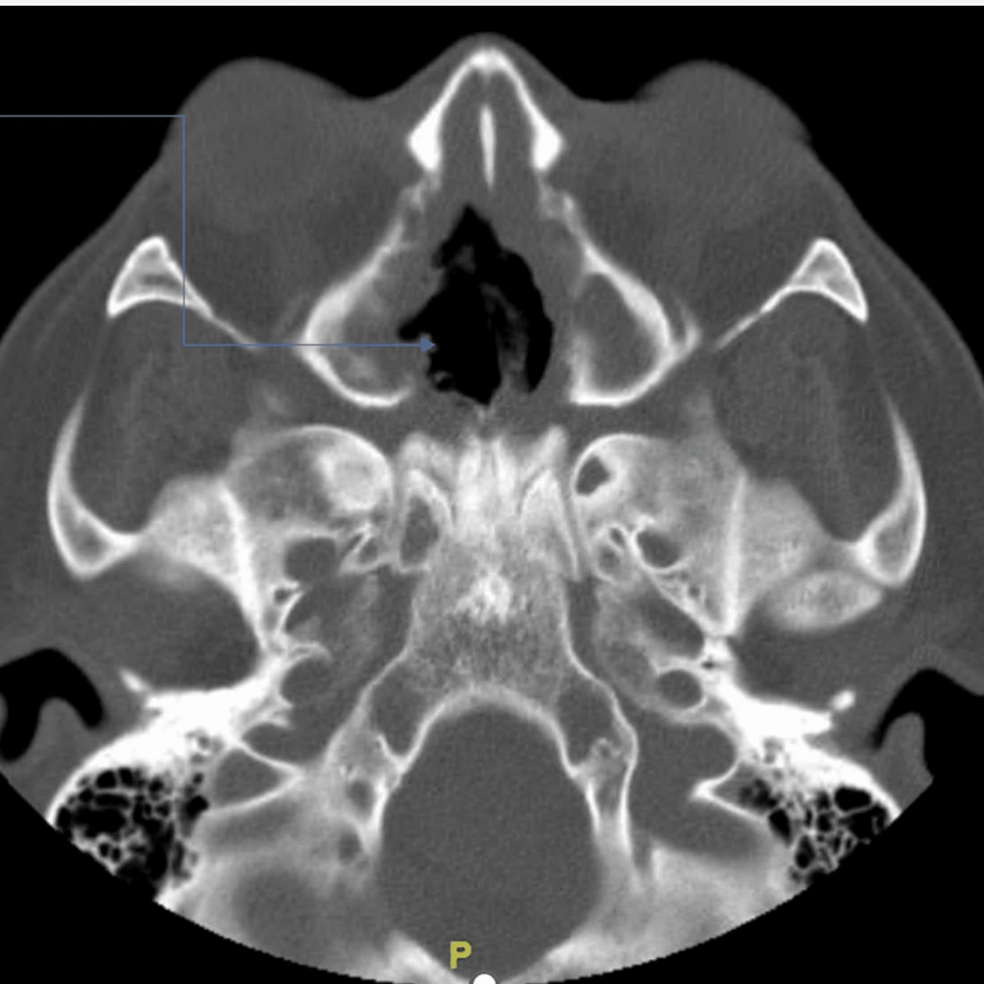
Extensive paranasal sinus disease with large nasal septal perforation, unchanged from prior (as shown in arrow).

Due to concerns regarding significant nasal and septal erosion, along with a suspected oroantral fistula, empirical treatment was initiated with vancomycin, zosyn, and liposomal amphotericin B. Subsequent cultures were performed, revealing the presence of a mild growth of Candida glabrata, Candida dublinesis, and minimal growth of Klebsiella ozaenae. Moreover, ENT and infectious disease were consulted. A subsequent nasal cavity endoscopy was conducted, unveiling notable crusting and ulceration of the septal perforation and erosion within the nasal cavity. The ulcerated mucosa was covered with a copious amount of purulent discharge. The tissue was biopsied. The endoscopy findings confirmed the existence of an oronasal fistula. Initial differentials included Wegner's granulomatosis, rhinoscleroma, sarcoidosis, vasculitis, fungal infection, and IgG4-related disease. Rheumatology was consulted and recommended further investigation for autoimmune diseases, such as testing for antinuclear antibodies (ANA) to evaluate for systemic lupus erythematosus (SLE), immunoglobulin G subclass 4, and Sjogren's antibody., however, all the autoimmune workup was negative including ANCA, Anti-DNA negative, C3/C4 negative, ANA positive with titers of 1:40 with a nuclear homogenous pattern. Furthermore, the patient’s chest x-ray did not show any cavitary lesions and her urinalysis did not reveal any hematuria, which made Wegner’s granulomatosis more unlikely. IGG4 antibodies come back positive with levels elevated at 150.8mg/dl. Moreover, a serial CT scan of facial bones without contrast showed extensive paranasal sinus disease with large nasal septal perforation, as shown in Figure 2. 

**Figure 3:**
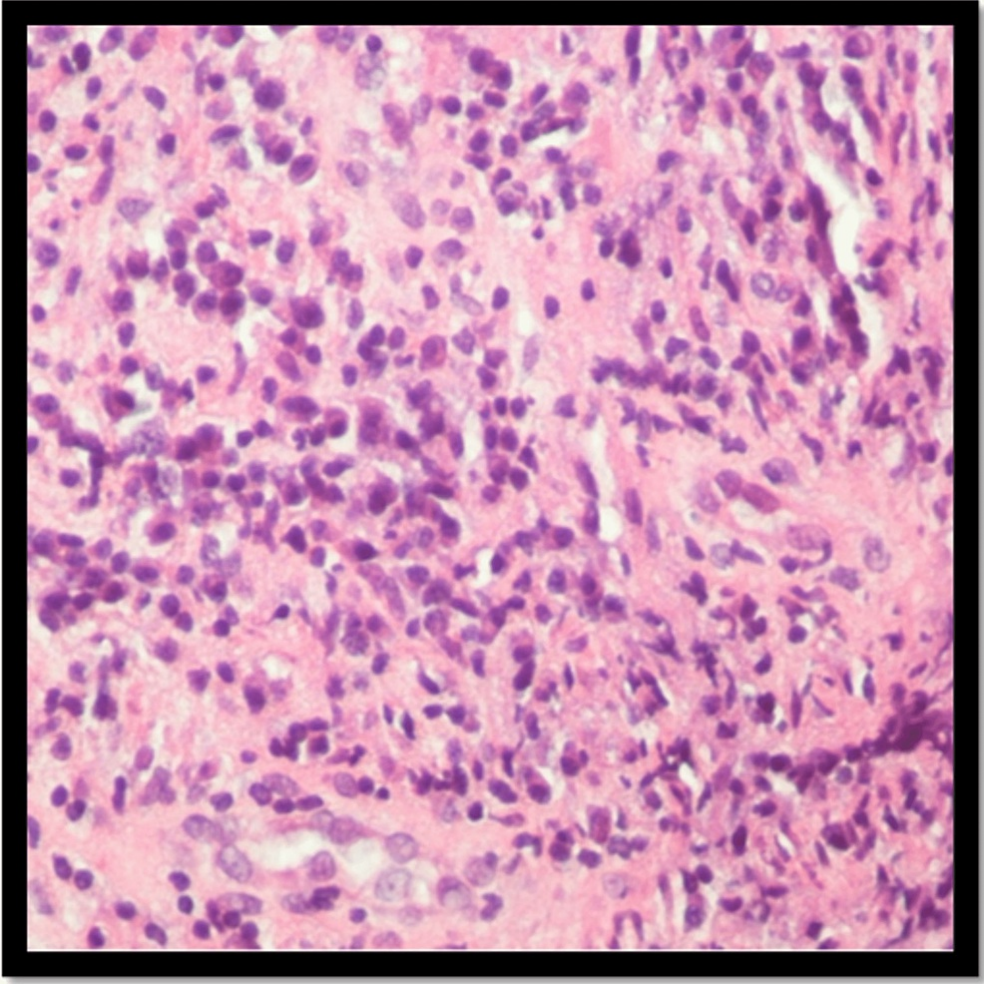
Fragments of squamous epithelium with ulcer, granulation tissue, and fibrinoinflammatory debris. The inflammatory infiltrate is mixed but includes mostly neutrophils and plasma cells. Eosinophils are rare.

**Figure 4:**
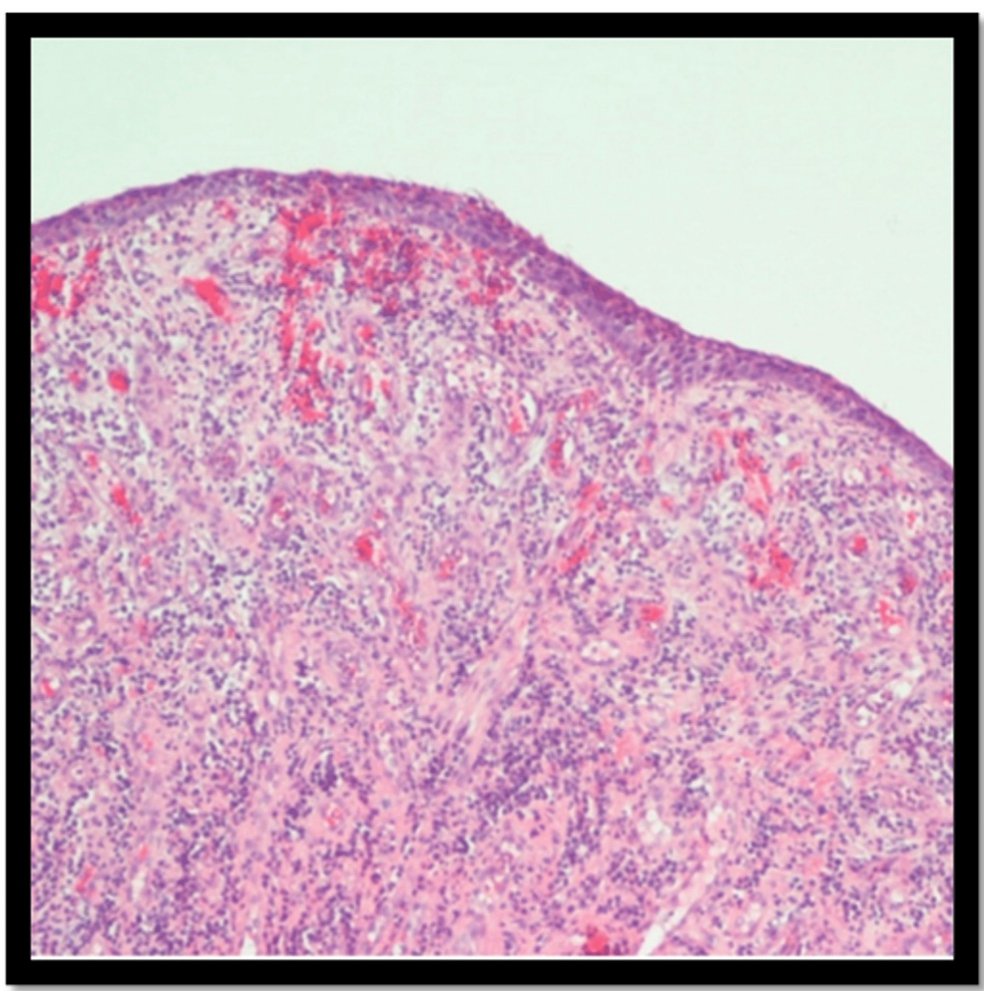
Benign squamous mucosa and submucosa showing necrosis, ulcer, marked acute and chronic inflammation with lymphoplasmacytic aggregate, and granulation tissue reaction.

**Figure 5:**
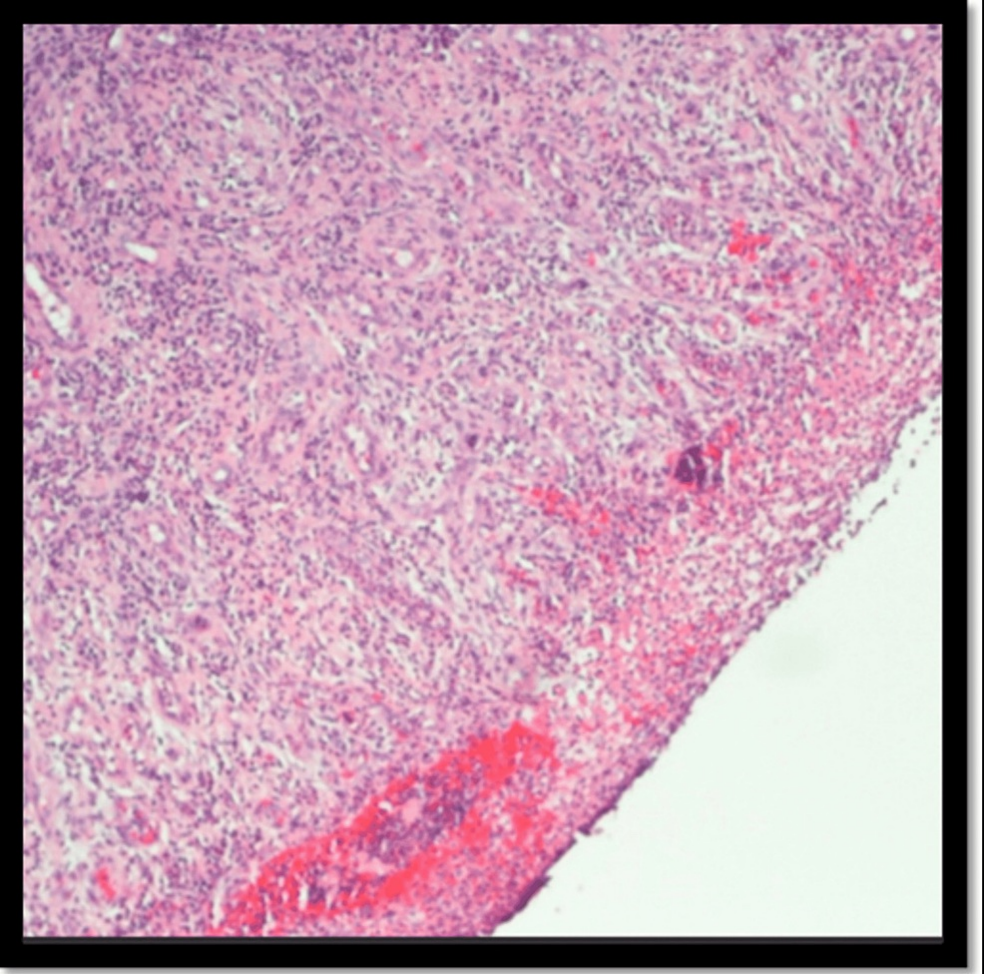
The inflammatory infiltrate is mixed but includes mostly neutrophils and plasma cells. IgG and lgG4 immunostain is performed and the IgG4 to IgG ratio is approximately 15-20%. lgG4 staining is present in occasional plasma cells and focally reaches greater than 60 plasma cells in one hpf.

Further biopsy analysis reported an inflammatory infiltrate characterized by a combination of cells, primarily neutrophils and plasma cells, with occasional eosinophils. A periodic acid-Schiff (PAS) stain was conducted to detect fungal organisms, but none were found. Immunostaining was performed to assess IgG and IgG4, showing an IgG4 to IgG ratio of approximately 15-20%. (Figures 3, 4, 5). 

Antibiotics were deescalated to ceftriaxone 2 grams once a day and oral metronidazole for six weeks as blood cultures were negative. The patient responded well to the antibiotics without the need for any steroids. She was discharged with outpatient follow-up appointments with Infectious diseases, Rheumatology and ENT for further management and follow-up for IgG4-related disease.

## Discussion

IgG4-related disease is known to impact multiple organ systems, with it commonly associated with type 1 autoimmune pancreatitis and IgG4-related sclerosing cholangitis [6]. This case report presents a patient with an atypical presentation of IgG4-related disease, wherein the patient presented with an oronasal fistula. A literature search on PubMed yielded no reports of IgG4-RD with the development of an oronasal fistula, thus signifying its novelty. 

Diagnosing IgG4-RD is challenging based on clinical presentation due to the lack of typical findings [7]. This necessitates a biopsy of the affected region. Histopathological findings of this disease include the observation of dense polyclonal lymphoplasmacytic infiltrates of IgG4-positive plasma cells [8]. Additionally, storiform fibrosis, obliterative phlebitis, and possible eosinophilia may be observed [8]. The patient's laboratory findings indicate an IgG4-positive antibody count of 150.8 mg/dL, which is significantly higher than the normal range of 4-80 mg/dL and crosses the clinical threshold required for an IgG4-RD diagnosis [9]. 

Involvement of the skull in a confirmed case of IgG4-RD is relatively rare and can be compared to a 2015 case of IgG4-RD that showed a mass in the right nasopharynx, blocking the Eustachian tube and leading to symptomatic mastoiditis. The distribution of fibrosis was reminiscent of nasopharyngeal carcinoma and involved the base of the skull [10]. When comparing the two patients, an elevation of erythrocyte sedimentation rate (ESR) of 66 mm/h (ref range: 0-15 mm/h) and a C-reactive protein (CRP) level of 2.030 mg/dL (ref range: <0.8 mg/dL) were observed. Our patient had a relatively high CRP level of 23.52 mg/dL two days post-admission but then had decreased to an ESR level of 5.0 mm/h and a CRP value of less than 3.00 mg/dL while maintaining an IgG4 level of 150.8 mg/dL [10]. 

This patient was also found to have an infection of Klebsiella ozaenae, which presents with similar findings of foul-smelling nasal discharge, nasal congestion and crusting, anosmia, nasal dryness, epistaxis, and nasal deformities [11]. However, the indication to look for IgG4-RD in this individual is due to the resolution of the infection during her stay and a persistent increase in the IgG4-positive titer count. 

IgG4-RD has an undefined etiology, with some studies reporting a genetic association between patients with specific HLA and non-HLA subtypes being at a higher risk [12]. Other studies have also mentioned an association with exposure to certain solvents, oils, and dust particles in IgG4-related diseases of pancreatitis and cholangitis [13]. Patients presenting with retroperitoneal fibrosis have an associated risk factor of tobacco and asbestos exposure [14]. The patient presented in this report has a history of tobacco intake. 

IgG4-RD has frequently been shown to cause dense fibrosis in the affected tissues, such as eosinophilic angiocentric fibrosis, which presents with nasal obstructive symptoms [15]. Conversely, the disease is also recorded to present with the development of a cholecystocutaneous fistula [16]. This highlights the need to differentiate between possible diagnoses of fistulas in patients with an unremarkable medical history. 

There are multiple possible renal manifestations of IgG4-related disease, including membranous glomerulopathy, tubulointerstitial nephritis, and obstructive nephropathy. Our patient has a history of CKD which may have been the result of chronic underlying, undiagnosed, IgG4-related disease-based fibrosis and lymphoplasmacytic infiltrate [17]. Our patient did not exhibit signs of urinary retention and thus fibrosis leading to urinary outflow tract is unlikely, however, a dedicated renal biopsy with histopathological review in the future would be helpful in connecting her CKD with IgG4-related disease. In this case, the presence of fibrosis with lymphocyte predominance with high IgG4-positive cells would support this diagnosis [18].

Treatment for IgG4-RD depends on the clinical severity of the patient at the time of diagnosis. All symptomatic patients must be treated with glucocorticoids, with the option of an additional immunosuppressive agent such as rituximab [19]. Asymptomatic patients are treated with this therapy only if the disease is progressive in nature [20]. The patient in this report exhibits a progressive presentation of the disease with an increasing IgG4 count, making her a suitable candidate for the medication. 

## Conclusions

In conclusion, IgG4-related disease (IgG4-RD) is an immune-mediated fibroinflammatory condition that affects multiple organs and is characterized by a wide range of clinical presentations and diagnostic challenges. Patients typically experience a gradual emergence of an organ-specific mass or enlargement of the affected organ. This condition predominantly affects middle-aged and elderly males but can also occur in females, and pediatric cases have been observed. Measuring the concentration of IgG4 antibodies in the bloodstream is a valuable indicator for diagnosing and predicting IgG4-related disease (IgG4-RD). Glucocorticosteroids are the initial treatment for IgG4-related disease (IgG4-RD), and immunosuppressive medications can be beneficial if glucocorticoids alone are inadequate or cannot be successfully tapered off. The prognosis of IgG4-related disease (IgG4-RD) varies depending on the organs involved and the timeliness of diagnosis and treatment. Early identification and proper management can lead to positive outcomes, including improved organ function and quality of life. However, it is important to note that some patients may experience relapses or irreversible organ damage, emphasizing the significance of long-term monitoring and follow-up care. 
